# Method Validation and Measurement Uncertainty Estimation for Determination of Multiclass Pesticide Residues in Tomato by Liquid Chromatography-Tandem Mass Spectrometry (LC-MS/MS)

**DOI:** 10.1155/2024/3846392

**Published:** 2024-01-10

**Authors:** Suraj Shrestha, Bandana Lamichhane, Nibedita Chaudhary

**Affiliations:** National Food and Feed Reference Laboratory, Department of Food Technology and Quality Control, Kathmandu, Nepal

## Abstract

Method validation is an essential technique for ensuring the reliability and accuracy of an analytical method. This study aimed to optimize and validate a fast, reliable, and accurate method for quantitatively determining pesticide residues of diverse chemical classes in the tomato matrix. Various method performance characteristics were tested and compared with predefined criteria. Twenty-six different pesticides of diverse chemical classes were selected based on their use in tomato cultivation and the availability of reference materials. The pesticide residues in tomato samples were extracted with the QuEChERS technique with some modifications, followed by injection into an LC-MS/MS system operating in an optimized method. The validated method demonstrated reasonable specificity, as there were no interferences from matrix components at the retention times of pesticides. The calibration curves for all pesticides exhibited excellent linearities, with correlation coefficients exceeding 0.99. No significant matrix effect was observed for all pesticides in tomatoes, as the values fell within the range of ±20%. All pesticides were quantified successfully at a concentration of 5 *μ*g/kg except for carbaryl, with an average recovery of more than 70% and a relative standard deviation of less than 20%. Similarly, measurement uncertainties were also estimated based on the validation data, and the values were found below the default limit of 50%. Subsequently, the validated method was applied to analyze 52 locally collected tomato samples. Study findings revealed that only four of the studied pesticides were detected in these samples, and their concentrations were below the maximum residue limits (500 *µ*g/kg each for carbendazim, imidacloprid, and metalaxyl) established for tomatoes by the Government of Nepal and the Codex Alimentarius Commission.

## 1. Introduction

Pesticides are widely used in agricultural production, including in the cultivation of tomatoes. Several groups of pesticides are used to manage weeds, insects, and other pests. However, some pesticides persist as residues in agricultural produce, and upon consumption of such contaminated produce, these pesticide residues enter the human body, posing potential adverse health impacts [1, 2]. Due to the hazardous nature of these pesticides, their presence is closely monitored in food to inhibit the potential adverse effects on human health. Consequently, many countries have established the maximum residue limits (MRLs) for the residues in various foods. Thus, monitoring the level of pesticide residues in food is desirable to ensure food safety.

Pesticide residue extraction from a food matrix is challenging due to its low concentration and potential interferences from the complex sample matrix [3]. Several sample extraction protocols are employed for this purpose [4]. However, the QuEChERS (Quick, Easy, Cheap, Effective, Rugged, and Safe) extraction technique is the obvious choice for multiresidue analysis due to its ease of use and fast extraction time [5–7]. Additionally, pesticides with a broad range of chemical nature can be extracted simultaneously. It involves two steps: sample extraction and sample cleanup. In the first step, residues are extracted from the homogeneous sample by adding acetonitrile and a blend of salts. The salt mixture allows the normally miscible organic solvent to separate from the water in the sample. In the second step, an aliquot of the organic phase from step 1 is taken for the cleanup through the use of dSPE (dispersive solid-phase extraction), where PSA (primary secondary amine) and anhydrous MgSO_4_ are used to remove residual water and many potential interfering substances such as sugar and organic acids from the extract. Based on the nature of the sample, other reagents, such as C18 and graphitized carbon black (GCB), can be added in the second step to facilitate the effective cleanup [8–11].

Several analytical techniques, such as gas chromatography-mass spectrometry, gas chromatography coupled to an electron capture detector, high-performance liquid chromatography, and liquid chromatography-tandem mass spectrometry, are available to monitor the level of pesticide residue in a food matrix [7, 12]. Among them, LC-MS/MS is one of the most widely used analytical techniques for pesticide residue analysis in food due to its ability to perform multiresidue analysis quickly with remarkable sensitivity. This study optimized an LC-MS/MS-based method, recognizing the necessity for a fast and reliable method for analyzing multi residue. Various method parameters were optimized to get an optimum response for each of the 26 selected pesticides. These pesticides belong to various chemical classes, including but not limited to carbamates, organophosphates, benzimidazoles, and neonicotinoids. The selection of pesticides in the study was based on the probable use in tomato cultivation and the availability of reference standards. Similarly, tomatoes were chosen as a matrix for this study due to their widespread consumption and availability of already established MRLs for pesticide residues. The Government of Nepal has set MRLs for 75 pesticides in tomatoes, the maximum number of pesticides regulated for any food category in the country. After optimization, the method was validated to assess its applicability. Method validation is an important technique that assesses an analytical method's suitability for its intended purpose [13]. In validation, various performance criteria are examined to determine the fitness of purpose, and the results are compared against the predefined criteria. If all the tested parameters met the predefined acceptance criteria, then the method is considered fit for purpose. It is essential for ensuring the reliability of the test results. Although several method validation protocols are available depending on the nature of the analytical method, the widely used protocol for pesticide residue analysis is the SANTE guideline [14].

## 2. Materials and Methods

### 2.1. Chemicals and Reagents

Individual 16 pesticide standards were obtained from Sigma Aldrich, and a mixed standard of 10 carbamate pesticides was purchased from Restek. HPLC grade of acetonitrile, methanol, formic acid, ammonium formate, and acetic acid and analytical grade of anhydrous magnesium sulfate and primary secondary amine (PSA) were purchased from local suppliers. The individual stock solution other than the mixed standard solution was prepared at a concentration of 1000 mg/L by dissolving in an appropriate amount of methanol. All standard solutions were stored in a refrigerator at 4°C before use.

### 2.2. Instrumentation

The study was performed in an Agilent 1290 Infinity LC system connected to an Agilent 6460 triple quadrupole mass spectrometer equipped with Agilent Jet Stream electrospray ionization (AJS-ESI). The chromatographic and mass spectrometric data were acquired and analyzed by using MassHunter software. The chromatographic separation of pesticides was carried out in an Agilent Poroshell 120 EC-C18 analytical column having dimensions of 3.0 × 50 mm and 2.7 *μ*m particle size.

### 2.3. Method Parameters

The method parameters were optimized before starting actual validation. Separation was carried out in a gradient mode with mobile phase A consisting of 0.1% formic acid and 5 mM ammonium formate in water. In contrast, mobile phase B comprised the same composition of formic acid and ammonium formate in methanol. The gradient started with 5% of mobile phase B, remaining constant until 0.5 minutes. Subsequently, it increased linearly to 65% at 5 minutes and rose to 95% at 6.5 minutes, maintaining the same composition until 9.0 minutes. At 9.1 minutes, the value of mobile phase B decreased sharply to 5% and remained the same until 12 minutes. The optimum flow rate, column temperature, and injection volume were 0.5 mL/min, 40°C, and 3 *μ*L, respectively. Each analysis was completed in 12 minutes.

Pesticides were ionized in positive electrospray ionization (ESI) mode and acquired in dynamic multiple reaction monitoring (dMRM) mode. The optimized flow rates for drying gas and sheath gas were 10 L/min and 11 L/min, respectively, with their temperatures maintained at 250°C and 350°C, respectively. The nebulizer gas pressure was constant at 40 psi, while capillary and nozzle voltages were set at 4000 V and 300 V, respectively.

### 2.4. Sample Preparation

Pesticide residues incurred in tomato samples were extracted using the QuEChERS AOAC 2007.01 protocol [15] with some modifications. Homogenized samples underwent extraction using a solution comprising 1% acetic acid in acetonitrile. The phase separation of acetonitrile and water layers was achieved using a mixture of anhydrous magnesium sulfate and sodium acetate. The acetonitrile layer containing the extracted pesticides was cleaned using a primary secondary amine and anhydrous magnesium sulfate mixture. Generally, the final extract would undergo evaporation followed by reconstitution in an appropriate solvent that matches the mobile phase composition. However, in this study, the final extract was diluted with water in a 1 : 3 ratio, bypassing the time-consuming evaporation step.

### 2.5. Method Validation

Method validation is a process of demonstrating the fitness of a method for its intended purpose by examining and providing objective evidence [13]. The method was validated as per analytical quality control and method validation procedures for pesticides in food and feed [14]. Critical method performance parameters such as specificity, linearity, limit of quantification, trueness, and precision were rigorously assessed experimentally in a blank tomato matrix. The obtained data were compared against the predefined criteria outlined in the protocol.

### 2.6. Measurement Uncertainty

Every measurement is associated with a dispersion known as measurement uncertainty (MU). It gives a range of values within which the true value of the measured quantity is expected to lie. It reflects the inherent variability in a measurement process. In pesticide residue analysis, measurement uncertainty is critical during compliance statements against a standard. Measurement uncertainty values were estimated using a top-down approach based on the validation data [16, 17]. This approach uses trueness and precision data generated in the method validation experiment to estimate the MU value.

## 3. Results and Discussion

### 3.1. Optimization of Analyte-Dependent Mass Parameters

**Figure 1:**
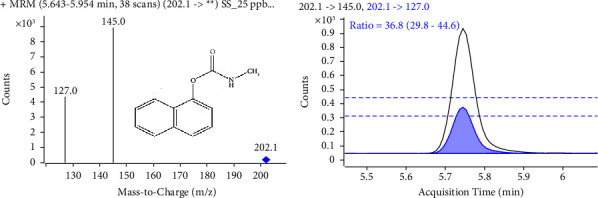
Mass spectra of carbaryl showing collision-induced dissociation of precursor [M + H]^+^ ion (m/z 202.1) into m/z 145.0 and m/z 127.0 (a) and the ion ratios of transition from 202.1 to 145.0 and 202.1 to 127.0 (b). The ion ratio for two transitions in carbaryl was found to be between 29.8 and 44.5, indicated by the two dotted lines, and this was also used to identify carbaryl in a sample accurately. Similarly, the more intense transition, 202.1 to 145.0, was used as the quantifier ion for carbaryl.

The mix working standard solution of all the pesticides was prepared at 1000 ng/mL and used to optimize their precursor ions, product ions, fragmentor, and collision voltages. The optimization work was performed by injecting pesticide standard solution without a column, and data were acquired using MassHunter Optimizer software. All pesticides showed good response in positive ESI mode, with the majority forming protonated ions [M + H]^+^, except for aldicarb, aldicarb sulfone, and propargite, which formed ammonium adducts [M + NH_4_]^+^ as a precursor ion. The optimized fragmentor voltages were found in the range of 50–145 V for all precursor ions, while collision energies ranged from 0 to 48 V for all product ions. Two product ions per precursor ion, their ion ratios, and retention time were utilized for pesticide confirmation [14]. The mass spectra and the ion ratio of carbaryl are shown in Figure 1. Initially, pesticides were monitored in multiple reaction monitoring (MRM) mode to obtain the retention times of each pesticide. Subsequently, it was upgraded to dynamic multiple reaction monitoring (dMRM) mode for maximizing sensitivity. In dMRM mode, specific MS transitions are monitored in a narrow time window corresponding to their expected elution from LC rather than throughout the full analysis time. The transition from MRM to dMRM drastically enhances the sensitivity of pesticides, as the MS duty cycle is not wasted by monitoring them when they are not expected to elute from LC [18]. The optimum acquisition parameters of all the target pesticides are summarized in Table 1.

### 3.2. Optimization of Chromatographic Parameters

**Figure 2:**
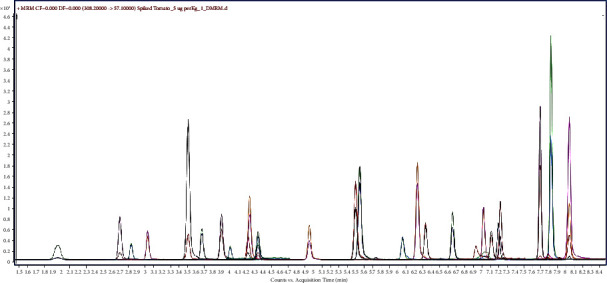
Extracted ion chromatogram (EIC) of the tomato blank sample spiked at 5 *μ*g/kg (vial concentration 1.25 ppb for each pesticide, except carbaryl) and on column mass of 3.75 picogram of each pesticide. Each peak comprises two MRM transitions (precursor to product ion 1 and precursor to product ion 2) monitored for a pesticide.

Various mobile phase gradient programs were studied in search of optimal resolution and sensitivity. However, due to the diverse chemical nature of multiresidues, some peaks were coeluted even after numerous experiments. The extracted ion chromatogram of mixed standard pesticides in an optimum method (presented in Section 2.3) is shown in Figure 2.

### 3.3. Modification of Sample Preparation

This study followed the QuEChERS AOAC 2007.01 protocol [15] without deviation until the cleanup step. However, the solvent evaporation of the final extract was skipped to shorten the sample preparation time. The evaporation processes involving a nitrogen turbo evaporator are time consuming and involve the risk of degradation of certain pesticides if temperature is not maintained carefully. An alternative approach was employed to avoid the probable degradation of pesticides and reduce the lengthy evaporation time. In this approach, the final acetonitrile extract was diluted with water in a ratio of 1 : 3 instead of the final evaporation step. This dilution considerably shortened the sample preparation time while ensuring the solvent matching with the mobile phase, resulting in satisfactory peak shapes, especially for early eluting pesticides.

### 3.4. Method Validation

#### 3.4.1. Specificity

**Figure 3:**
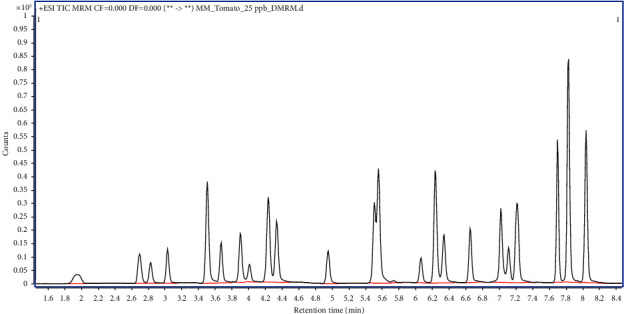
Overlaid total ion chromatograms (TICs) of 25 ng/mL matrix-matched standard solution (black) and tomato blank (red).

The specificity of an analytical method is examined to verify the absence of potential interfering compounds at the retention time of the target analytes. The tomato blank matrix and matrix-matched standard solutions were analyzed simultaneously to assess the specificity of the method. Comparison of total ion chromatograms (TICs) of both samples, as shown in Figure 3, revealed the absence of any significant interfering peaks at the retention times of all target pesticides, thus indicating the specificity of the method.

#### 3.4.2. Linearity

**Figure 4:**
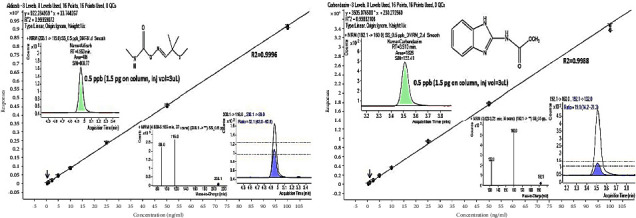
Calibration curves of aldicarb (a) and carbendazim (b).

Linearity was evaluated from the calibration curve constructed from a series of eight duplicate concentrations ranging from 0.5 ng/mL to 100 ng/mL in a solvent with a final composition of 1 : 3 acetonitrile and water. The calibration curves were best fitted to a linear curve with weight 1/x. Good linear relationships were observed with regression coefficients (*R*^2^) of 0.99 or higher across the examined concentration range for all the pesticides. Calibration curves of some selected pesticides are presented in Figure 4, while regression coefficients (*R*^2^) and slopes of all the pesticides are summarized in Table 2.

#### 3.4.3. Matrix Effect

The matrix effect is frequently encountered in LC-MS/MS analysis due to ion suppression or enhancement effect. It arises from coextracted compounds from the sample matrix, influencing analyte concentration measurement [19]. If not adequately compensated for, the matrix effect can significantly affect the trueness of analytical results. The matrix effect was evaluated by comparing the slopes of the solvent standard (SS) calibration curve with the slopes of the matrix-matched (MM) calibration curve, constructed using standards prepared in a blank sample matrix [20]. The percentage matrix effect was determined using the following formula [21]:

(1) $$\begin{array}{c} \text{matrix}\;\text{effect}\;\left(\%\right)=\frac{\left(\text{slope}\;\text{of}\;\text{MM}\;\text{curve}-\text{slope}\;\text{of}\;\text{SS}\;\text{curve}\right)*100}{\text{slope}\;\text{of}\;\text{SS}\;\text{curve}}. \end{array}$$

Matrix effect values between 0 and 20% are considered low matrix effects, and the correction for the matrix effect is not necessary. However, if more than a 20% matrix effect is observed, it should be addressed to ensure more accurate results [14]. The negative value represents ion suppression, whereas the positive value represents ion enhancement. The matrix effects for all the analyzed pesticides were found within ±20%, as shown in Table 2, indicating the absence of significant matrix effects caused by the tomato matrix. In such instances, solvent standards could be used for quantifying pesticide residues incurred in unknown samples. This is particularly useful in routine analysis when no blank tomato matrix is available for preparing matrix-matched calibration standards.

#### 3.4.4. Limit of Quantification (LOQ)

The limit of quantification (LOQ) is a critical performance characteristic for pesticide residues. The value should be at or below the maximum residue level (MRL) to draw meaningful conclusions about pesticide residues incurred in a sample. LOQ can be determined using various methods [22, 23]. LOQ was determined by spiking a series of low concentrations in a blank tomato matrix. All pesticides, except carbaryl, were successfully analyzed at the 5 *µ*g/kg level, meeting all identification, recovery, and precision criteria. For carbaryl, it was 10 *µ*g/kg. These LOQs were much lower than the MRLs established for tomatoes by the Government of Nepal [24] and the Codex Alimentarius Commission [25]. The obtained values of LOQ are summarized in Table 2.

#### 3.4.5. Trueness and Precision

Various approaches, such as recovery experiments and analysis of certified reference material, have been employed to assess an analytical method's trueness [13, 26]. In this study, trueness was evaluated by recovery experiments, in which blank tomato samples were spiked at three different concentrations: low (5 *µ*g/kg), medium (10 *µ*g/kg), and high (40 *µ*g/kg), each level in 6 replicates. The recoveries in the 70–120% range were obtained, exhibiting good trueness of the method. Similarly, precision was assessed as repeatability and within-laboratory reproducibility. Both were below 20% relative standard deviation (RSD) for all pesticides. The obtained values of trueness and precision are summarized in Table 3.

#### 3.4.6. Measurement Uncertainty (MU)

Measurement uncertainty (MU) is inherently associated with any measurement. There are various approaches available for the estimation of measurement uncertainty. The most widely used approach is the ISO GUM approach [22, 27, 28]. However, estimating using the bottom-up approach for multiresidue is impractical. Thus, the present study estimated MUs using a top-down approach. Intralaboratory validation data were used to estimate the standard uncertainty and then expressed at a 95% confidence level. Two significant sources, precision and trueness (as bias), were taken as the main contributors to measurement uncertainty. The standard uncertainty of each pesticide was estimated using the following formula:

(2) $$\begin{array}{c} u=\sqrt{u{\left(\text{bias}\right)}^{2}+u{\left(\text{precision}\right)}^{2}}. \end{array}$$

Uncertainty due to bias was estimated from the recovery experiment, while the percentage relative standard deviation of within-laboratory reproducibility was used to estimate the uncertainty arising from precision. The expanded uncertainty was calculated by multiplying the standard uncertainty with a coverage factor (*k*) of 2, which approximately gives a confidence level of 95%:

(3) $$\begin{array}{c} U=k\times u. \end{array}$$

**Figure 5:**
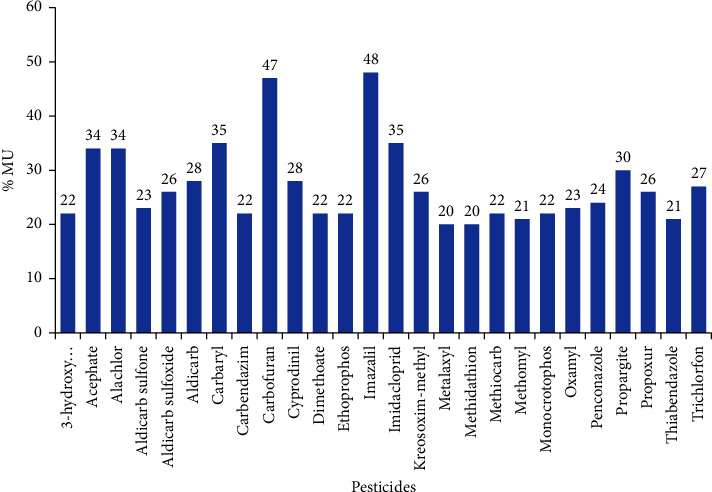
Measurement uncertainty (%) for each pesticide. The measurement uncertainties are expressed at a 95% confidence level.

The obtained values for all the target pesticides, as presented in Figure 5, were lower than the 50% default value employed by many regulatory authorities for enforcement decisions.

### 3.5. Application of the Validated Method to the Real Sample

**Figure 6:**
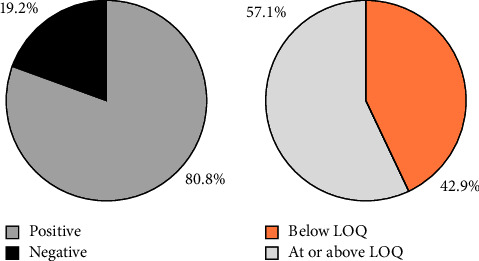
Pesticide residue status in (a) analyzed samples and (b) residue content in positively identified samples.

The validated method was further applied to 52 tomato samples collected from various local vegetable markets in Kathmandu, Nepal, for the simultaneous analysis of pesticide residues. The findings of the analyzed samples are shown in Figure 6. None of the target pesticides were detected in 10 (19.2%) samples. Pesticides were detected in 80.8% of the samples analyzed, ranging from one to four pesticides (carbendazim, imidacloprid, metalaxyl, and thiabendazole). Carbendazim, imidacloprid, metalaxyl, and thiabendazole were detected in 34, 28, 7, and 1 samples, respectively, either individually or in combination. Of 42 positive samples, 24 (57.1%) samples were found to contain at least one pesticide residue at or above the LOQ level, while in the remaining 18 (42.9%) samples, pesticides were found below the LOQ level for each detected pesticide. The concentrations of pesticide residues in analyzed tomato samples were found to be in the range of 8–268 *µ*g/kg, with an average of 63 *µ*g/kg for carbendazim, 5–125 *µ*g/kg, with an average of 34 *µ*g/kg for imidacloprid, and 8–67 *µ*g/kg, with an average 32 *µ*g/kg for metalaxyl. These pesticide residue concentrations were significantly lower than the maximum residue limit (MRL) set by the Codex Alimentarius Commission [25] and the Nepal Government [24] for tomatoes (500 *µ*g/kg for each of the pesticides mentioned above). Similar results were observed in a previous study conducted by Bhandari et al. [29] in selected tomato samples (*N* = 32) from Nepal.

## 4. Conclusions

Pesticide residues in food have always been a public concern, with many people aware of harmful health effects caused by consuming food containing these residues. Analyzing the food samples for pesticide residues is essential to safeguard public health. Routine analysis for residues requires a quick and accurate method with high sensitivity. Thus, we optimized and validated an LC-MS/MS method to analyze 26 pesticide residues in tomato samples. All method validation performance characteristics were satisfactory, indicating the method's reliability.

Furthermore, MUs were also estimated, which would be helpful while deciding the compliance of tomato samples against the established MRL. The validated method was successfully applied to analyze actual tomato samples, and some positive samples were detected and subsequently quantified. Even though 80.8% of the analyzed samples (*N* = 52) had one to four target pesticide residues, these amounts were significantly lower than the MRLs set for tomatoes. The validated method offers several advantages, including simple extraction, cleanup without an additional evaporation step of the final extract, high sensitivity, and good accuracy. Additionally, more pesticides can be included in the validated method to cover a broader range of pesticides. It offers an efficient solution for a quality control laboratory that analyzes multiresidues in tomato samples.

## Figures and Tables

**Table 1:** Name, ion type, precursor ion, product ions, fragmentor voltage, collision energies, cell accelerator potential, retention time, and ionization polarity of target 26 pesticides.

S. N.	Pesticide	Ion type	Precursor ion (m/z)	Product ions^*∗*^(m/z)	Frag (V)	CE (V)	Cell acc (V)	Rt (min)	ESI polarity
1	3-Hydroxycarbofuran	[M + H]^+^	238.1	**181.1**	70	5	6	4.36	Positive
				163.1	70	5	6	4.36	Positive

2	Acephate	[M + H]^+^	184	**142.9**	50	4	6	1.98	Positive
				95	50	20	6	1.98	Positive

3	Alachlor	[M + H]^+^	270.1	**238**	90	4	6	7.07	Positive
				162.1	90	16	6	7.07	Positive

4	Aldicarb	[M + NH_4_]^+^	208.1	**116**	60	0	6	4.95	Positive
				89	60	12	6	4.95	Positive

5	Aldicarb sulfone	[M + NH_4_]^+^	240	**148**	80	5	6	3.03	Positive
				86	80	10	6	3.03	Positive

6	Aldicarb sulfoxide	[M + H]^+^	207.1	132	55	0	6	2.83	Positive
				**89.1**	55	8	6	2.83	Positive

7	Carbaryl	[M + H]^+^	202.1	**145**	130	4	6	5.76	Positive
				127	130	28	6	5.76	Positive

8	Carbendazim	[M + H]^+^	192.1	**160**	130	16	6	3.52	Positive
				132	130	32	6	3.52	Positive

9	Carbofuran	[M + H]^+^	222.1	**165**	75	4	6	5.57	Positive
				123	75	20	6	5.57	Positive

10	Cyprodinil	[M + H]^+^	226.1	93.1	130	36	6	7.12	Positive
				**77.1**	130	48	6	7.12	Positive

11	Dimethoate	[M + H]^+^	230	198.9	70	4	6	4.24	Positive
				**124.9**	70	16	6	4.24	Positive

12	Ethoprophos	[M + H]^+^	243.1	130.9	85	16	6	7.02	Positive
				**96.9**	85	28	6	7.02	Positive

13	Imazalil	[M + H]^+^	297.1	**158.9**	120	20	6	6.1	Positive
				69.1	120	16	6	6.1	Positive

14	Imidacloprid	[M + H]^+^	256.1	209	80	12	6	4.02	Positive
				**175**	80	12	6	4.02	Positive

15	Kresoxim-methyl	[M + H]^+^	314.1	267	60	0	6	7.23	Positive
				**116**	60	8	6	7.23	Positive

16	Metalaxyl	[M + H]^+^	280.2	220	90	8	6	6.24	Positive
				**192.1**	90	12	6	6.24	Positive

17	Methidathion	[M + H]^+^	303	144.9	65	4	6	6.33	Positive
				**85.1**	65	16	6	6.33	Positive

18	Methiocarb	[M + H]^+^	226.1	169	70	4	6	6.67	Positive
				**121**	70	12	6	6.67	Positive

19	Methomyl	[M + H]^+^	163.1	135	145	8	6	4.34	Positive
				**107**	145	20	6	4.34	Positive

20	Monocrotophos	[M + H]^+^	224.1	**193**	60	0	6	3.67	Positive
				126.9	60	12	6	3.67	Positive

21	Oxamyl	[M + H]^+^	220.1	163	110	4	6	4.34	Positive
				**107**	110	24	6	4.34	Positive

22	Penconazole	[M + H]^+^	284.1	158.9	115	28	6	7.21	Positive
				**70.1**	115	12	6	7.21	Positive

23	Propargite	[M + NH_4_]^+^	368.2	**231.1**	80	4	6	7.84	Positive
				175	80	12	6	7.84	Positive

24	Propoxur	[M + H]^+^	210.1	168	60	0	6	5.52	Positive
				**111**	60	8	6	5.52	Positive

25	Thiabendazole	[M + H]^+^	202	**175**	125	24	6	3.92	Positive
				131	125	36	6	3.92	Positive

26	Trichlorfon	[M + H]^+^	256.9	220.9	90	4	6	4.24	Positive
				**108.9**	90	12	6	4.24	Positive

^*∗*^Product ions used as quantifier ions are indicated in bold, while qualifier product ions are not in bold. Rt, retention time; CE, collision energy; Frg, fragmentor voltage; Cell acc, cell accelerator potential; ESI, electrospray ionization.

**Table 2:** Regression coefficient (*R*^2^), slope, matrix effect, method LOQ, and method range of the pesticides.

S. N.	Pesticide	Solvent standard calibration curve		Matrix-matched calibration curve		% matrix effect	Method LOQ (*µ*g/kg)	Method range (*µ*g/kg)
		*R* ^2^	Slope	*R* ^2^	Slope			
1	3-Hydroxycarbofuran	0.999	177.591	0.999	187.511	6	5	5–400
2	Acephate	1.000	797.581	0.999	783.193	−2	5	5–400
3	Alachlor	0.999	458.473	0.999	438.277	−4	5	5–400
4	Aldicarb sulfone	0.999	663.776	0.999	657.814	−1	5	5–400
5	Aldicarb sulfoxide	0.999	383.474	0.999	388.445	1	5	5–400
6	Aldicarb	1.000	922.285	0.999	864.694	−6	5	5–400
7	Carbaryl	0.998	59.901	0.998	50.906	−15	10	10–800
8	Carbendazim	0.999	3505.077	0.999	3320.299	−5	5	5–400
9	Carbofuran	1.000	2655.585	0.999	2394.499	−10	5	5–400
10	Cyprodinil	1.000	714.738	0.999	692.753	−3	5	5–400
11	Dimethoate	0.999	1627.152	0.999	1570.99	−3	5	5–400
12	Ethoprophos	0.999	1167.623	0.998	1125.412	−4	5	5–400
13	Imazalil	1.000	561.124	0.999	492.480	−12	5	5–400
14	Imidacloprid	1.000	270.316	1.000	325.353	20	5	5–400
15	Kresoxim-methyl	0.999	851.743	0.999	808.224	−5	5	5–400
16	Metalaxyl	0.999	2456.334	0.999	2413.506	−2	5	5–400
17	Methidathion	1.000	894.615	0.999	908.447	2	5	5–400
18	Methiocarb	0.999	1140.993	0.999	1141.137	0	5	5–400
19	Methomyl	1.000	578.232	1.000	576.797	0	5	5–400
20	Monocrotophos	1.000	779.925	0.999	790.445	1	5	5–400
21	Oxamyl	1.000	756.761	0.999	772.839	2	5	5–400
22	Penconazole	0.999	1371.04	0.998	1279.036	−7	5	5–400
23	Propargite	0.999	4822.576	0.999	4457.998	−8	5	5–400
24	Propoxur	1.000	2128.077	0.999	1817.774	−15	5	5–400
25	Thiabendazole	1.000	1222.917	1.000	1209.514	−1	5	5–400
26	Trichlorfon	0.994	501.997	0.999	601.180	20	5	5–400

Matrix effects with negative values indicate signal suppression, whereas positive values indicate signal enhancement.

**Table 3:** Trueness as % recovery and precision as % RSD of repeatability (within-laboratory reproducibility) of target pesticides in tomato matrix spiked at 5, 10, and 40 *µ*g/kg.

S. N.	Pesticide	Trueness			Precision as repeatability (within-laboratory reproducibility)		
		5 (*µ*g/kg) (*n* = 6)	10 (*µ*g/kg) (*n* = 6)	40 (*µ*g/kg) (*n* = 6)	5 (*µ*g/kg)	10 (*µ*g/kg)	40 (*µ*g/kg)
		% recovery			% RSD		
1	3-Hydroxycarbofuran	93	94	86	4.7 (8.2)	3.2 (4.8)	2.3 (1.7)
2	Acephate	81	80	81	2.4 (8.5)	2.2 (6.4)	1.0 (2.8)
3	Alachlor	89	90	87	3.5 (14.5)	3.6 (5.0)	1.2 (1.6)
4	Aldicarb sulfone	89	90	87	3.8 (8.3)	3.3 (4.9)	1.7 (1.6)
5	Aldicarb sulfoxide	87	88	83	3.6 (5.9)	3.5 (3.5)	1.4 (1.5)
6	Aldicarb	87	83	83	2.0 (8.7)	2.2 (6.5)	1.2 (2.2)
7	Carbaryl	^*∗*^	77	83	^*∗*^	6.1 (11.7)	5.0 (3.7)
8	Carbendazim	91	86	92	3.1 (9.9)	2.1 (8.3)	1.1 (2.0)
9	Carbofuran	87	85	79	2.5 (12.6)	2.8 (7.5)	2.4 (3.7)
10	Cyprodinil	90	85	83	5.6 (5.5)	3.9 (4.0)	1.1 (1.6)
11	Dimethoate	90	88	88	4.5 (7.0)	2.3 (4.8)	0.9 (1.3)
12	Ethoprophos	91	90	90	2.4 (7.4)	3.0 (4.1)	1.0 (1.2)
13	Imazalil	90	82	78	4.3 (13.3)	4.3 (8.6)	2.9 (4.6)
14	Imidacloprid	104	120	110	5.0 (6.9)	4.9 (4.1)	1.3 (4.6)
15	Kresoxim-methyl	90	90	86	2.5 (6.2)	3.4 (2.8)	1.0 (1.8)
16	Metalaxyl	91	90	89	2.9 (5.6)	2.4 (3.2)	1.2 (1.1)
17	Methidathion	90	93	88	3.2 (6.8)	4.8 (3.6)	1.8 (1.8)
18	Methiocarb	91	93	86	2.1 (4.7)	3.5 (3.1)	1.4 (1.6)
19	Methomyl	90	92	87	5.3 (6.7)	4.0 (4.7)	1.2 (1.9)
20	Monocrotophos	87	90	88	2.9 (6.8)	2.9 (3.2)	1.2 (1.1)
21	Oxamyl	89	93	86	3.9 (7.0)	3.7 (3.2)	1.7 (1.5)
22	Penconazole	88	87	87	3.0 (6.4)	3.2 (4.2)	0.7 (1.4)
23	Propargite	87	83	86	3.1 (5.4)	2.5 (4.5)	1.4 (2.0)
24	Propoxur	90	90	84	1.9 (4.7)	2.1 (1.8)	1.1 (1.3)
25	Thiabendazole	93	88	88	2.4 (6.5)	2.9 (5.1)	1.4 (1.3)
26	Trichlorfon	106	117	113	3.4 (8.2)	4.1 (4.5)	0.8 (3.4)

^*∗*^For carbaryl, a recovery experiment was performed at the 10 *µ*g/kg level.

## Data Availability

The experimental data used to support the findings of the study are included within the article.
